# Testing bird-driven diurnal trade-offs of the moon moth's anti-bat tail

**DOI:** 10.1098/rsbl.2022.0428

**Published:** 2023-02-01

**Authors:** Juliette J. Rubin, Nich W. Martin, Kathryn E. Sieving, Akito Y. Kawahara

**Affiliations:** ^1^ Department of Biology, University of Florida, Gainesville, FL 32611, USA; ^2^ McGuire Center for Lepidoptera and Biodiversity, Florida museum of Natural History, University of Florida, Gainesville, FL 32611, USA; ^3^ Entomology and Nematology Department, University of Florida, Gainesville, FL 32611, USA; ^4^ Department of Wildlife Ecology and Conservation, University of Florida, Gainesville, FL 32611, USA

**Keywords:** predator–prey, Saturniidae, Carolina wren, elaborate trait, evolutionary trade-offs

## Abstract

Traits are often caught in a dynamic tension of countervailing evolutionary pressures. Trade-offs can be imposed by predators evolutionarily curtailing the conspicuousness of a sexually selected trait, or acting in opposition to another natural selection pressure, for instance, a different predator with a divergent hunting strategy. Some moon moths (Saturniidae) have long hindwing tails that thwart echolocating bat attacks at night, allowing the moth to escape. These long tails may come at a cost, however, if they make the moth's roosting form more conspicuous to visually foraging predators during the day. To test this potential trade-off, we offered wild-caught Carolina wrens (*Thryothorus ludovicianus*) pastry dough models with real *Actias luna* wings that were either intact or had tails experimentally removed. We video recorded wrens foraging on models and found that moth models with tails did not experience increased detection and attack by birds. Thus, this elaborate trait, while obvious to human observers, does not seem to come at a cost of increased avian predator attention. The evolution of long hindwing tails, likely driven by echolocating predators at night, does not seem to be limited by opposing diurnal constraints. This study demonstrates the importance of testing presumed trade-offs and provides hypotheses for future testing.

## 1. Introduction

Animal traits are often shaped by a series of evolutionary trade-offs. Complex, conspicuous (i.e. elaborated) traits that provide benefits in one area of an animal's life may have costs in another. Trade-offs can be found among elaborated traits that have evolved in a sexual selection context, but that incur costs for the bearer via increased predator attention and attack. For example, bright colours in guppies [1], complex calls in Tungará frogs [2] and bioluminescent displays in fireflies [3] are all preferred by both choosing conspecific females and hunting predators.

Trade-offs can also occur solely among natural selection forces, when different predators and sensing systems create countervailing pressures on the same traits. For instance, iridescence in beetles may enhance camouflage against vertebrate predators [4], while increasing salience for invertebrate predators [5]. Similarly, *Bicyclus anynana* butterflies have a dry season and wet season morph with more conspicuous and duller eyespots, respectively. This polyphenism appears to be driven by the differential anti-predator efficacy of eyespots against vertebrate and invertebrate predators, where more conspicuous eyespots (wet season) thwart mantid predators and duller eyespots (dry season) prevent detection from birds [6].

Shifting predator communities emerge not just across seasons, but also within the span of a day. The cryptic colour of a mouse does not hide its footsteps from the discerning ears of an owl [7] and tiger moths that announce their chemical defence to bats via ultrasonic clicks at night must also advertise their noxiousness with bright colours to bird predators during the day [8]. Saturniid moths are an earless, non-sound-producing family of moths that live a limited time in adult form (approx. 7 days or less), during which they mate but do not feed [9]. Some saturniids have evolved long, twisted and cupped tails that spin behind the moth as it flies, thwarting bat attack. These tails have originated multiple independent times and function to draw predator strikes away from the body and toward these non-essential appendages [10,11]. While tails provide a benefit at night, they may pose a risk during the day by increasing the visual conspicuousness of a roosting moth.

Visual predators often rely on search images to detect cryptic prey among convoluted backgrounds [12,13]. While it seems likely that a moth with tails might attract a bird's attention with its odd shape, it is also possible that these tails might break up typical Lepidoptera search images, allowing moths to evade detection [14]. Using *Actias luna* moth models with and without tails, we tested the effect of elaborate hindwing tails during the day. We hypothesized that *A. luna* moths with tails would make the roosting moth more obvious to foraging birds, creating an evolutionary trade-off between the benefit a tail provides against nocturnal predators and the cost it incurs against diurnal predators.

## 2. Materials and methods

### (a) Experimental animals

To test the role of diurnal predation on hindwing tails, we used 23 Carolina wrens (*Thryothorus ludovicianus*), captured via mist netting and individually housed in outdoor flight cages at the USDA APHIS facility in Gainesville, FL, USA. Carolina wrens and *A. luna* co-occur in this region of Florida. Moreover, previous work indicates both that *A. luna* are palatable [15], and that wrens forage among leaves for prey and consume adult Lepidoptera [16]. Wrens also keep well in captivity and are food motivated. We gave all birds 2 full days following capture to acclimate to their new surroundings. They had access to water and dry feed *ad libitum* from an elevated platform in their cage, and once a day we provided them with live mealworms in their feeding tray on the platform. On acclimation days, we introduced a moth model body (described below) into the feeding trays to help birds identify this novel object as a food item. Birds were enrolled in a different feeding choice study involving presentations of other types of lepidopteran models on days either preceding or following this experiment. Given that both studies were non-invasive, and used acclimation periods and similar set-ups, we are confident there were no carryover effects. To test this, however, we included experiment sequence as a parameter in our models. We tested 26 birds in total, but three refused to strike either moth model on either day, and were therefore excluded from analysis. Permits related to this study (with K.E.S. as PI) included: USGS Federal Bird Banding Permit no. 22541 and Florida Fish and Wildlife Conservation Commission permit no. LSSC-20-00022 (for capture and temporary captivity); University of Florida IACUC protocol no. 201910895 and USDA APHIS protocol no. QA-3188 (for standards of care while in captivity). Birds were released back into the wild after completing all experimental trials.

### (b) Experimental design

**Figure 1.**
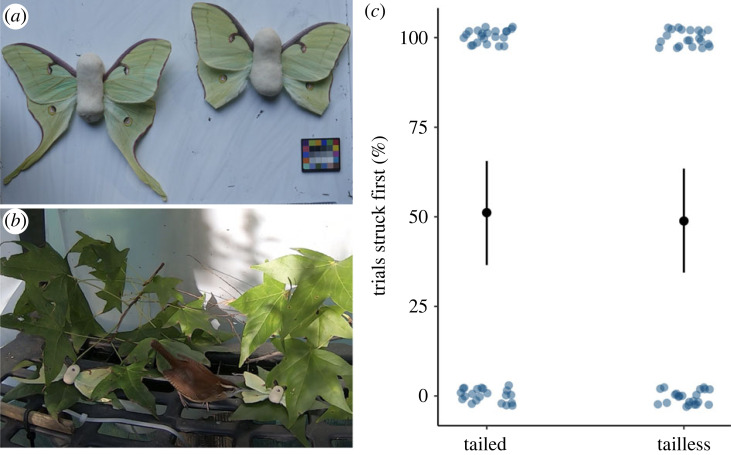
Hindwing tails in *Actias luna* moths do not come with a trade-off against foraging birds. (*a*) We pit Carolina wrens (*Thryothorus ludovicianus*) in outdoor flight cages against two different moth pastry dough models, using real *A. luna* wings. The treatments were either natural wings (tailed) or wings with the tails cut-off (tailless). (*b*) We presented these models on branches of sweetgum, *A. luna's* preferred host, and measured which model the bird attacked first. (*c*) Birds did not preferentially strike either treatment first. Thus, tails do not seem to draw avian predator attention, nor do they afford roosting moths safety by breaking search image. Central points depict mean marginal effects and error bars are 95% confidence intervals.

We presented each wren with two moth models simultaneously—one with tails and one without (figure 1*a*). To create moth model bodies, we wrapped two mealworms in a thin packaging of pastry dough (1/4 lard, 1/4 water and 1/2 white flour), following the method of Carroll & Sherratt [17]. We removed wings from dead, frozen *A. luna* specimens and inserted them into the pastry dough body. We determined which wing set would remain intact (treatment = tailed) versus tails cut-off (treatment = tailless) via coin toss. To account for any size differences between treatments, we took size-calibrated photos to calculate surface area and incorporated these surface area measurements into our statistical models. Using a nail driven through the pastry dough body, we affixed each model to a leaf on separate branches of sweet gum (*Liquidambar styraciflua*)—the luna moth's preferred host plant [18]. Prior to trial commencement, we food-deprived birds for 1 h. We recorded all interactions using a GoPro Hero 7 Black (2.7 K, 30 fps) and gave each bird 2 h to complete its trial. To test birds' initial detection responses, rather than captive learning, we ran individuals through only two trials, over two sequential days. We randomly selected the side of the platform on which to present each moth model on day 1 and then reversed positions on the day 2. Comparing results from the 2 days allowed us to determine whether birds were consistently attacking models on the same side of the platform, regardless of treatment (which would indicate a locational bias), or whether birds were consistently striking the same treatment of moth (which could indicate innate detectability biases or that experience from day 1 informed day 2). We arranged branches such that neither model was completely exposed or hidden, but that a portion of their wing area was bisected by leaves to create a more convoluted visual scene (figure 1*b*; electronic supplementary material, videos S1 and S2). To determine which moth model treatment the bird first attacked, we reviewed videos using VLC media player. We determined first attack by noting which model the bird first contacted with its beak (electronic supplementary material, videos S1 and S2).

### (c) Statistical analyses

We analysed our data using the ‘lme4’ [19] package in R studio. To account for a locational bias or a treatment bias, we built two separate sets of models. The first set included a parameter for which moth treatment was struck first as the response variable. Moth surface area, whether the bird attacked the treatment on the same side of the platform in its two consecutive trials, and whether the bird was used in this or another experiment first (to control for any effects this may have had) were included as fixed effects, with a random effect of cage in which the trial was performed. The second model set included the same parameters, with the exception of the side of platform that the bird attacked, which we substituted for a binomial parameter indicating whether the bird first attacked the same treatment of moth in its two consecutive trials. To measure moth surface area, we extracted the surface area of all four wings using the polygon tool in ImageJ [20]. We checked model goodness of fit using the ‘performance’ package [21]. See electronic supplementary material for model structures and fit.

To further test for any differences between bird attack on tailed and tailless models, we ran a set of counterfactual simulations using a Bayesian generalized linear mixed model, with a Bernoulli distribution and an inverse logit link function. These models are termed counterfactual because they estimate an event outcome from a probability distribution based on the observed data, to infer the effect of variable combinations that may not have occurred in the observed data. In the case of this experiment, the event is predatory attack and the treatment is the removal of *A. luna* moth tails. By randomly sampling across the range of possible confounding variable values (moth surface area, day of trial), we can block their influence on the predicted effects of the variables of interest, allowing for better inference. Moreover, by using the same randomly sampled confounding variable values for both treatments, we can create vectors of the predicted effects for each treatment and take their difference at each point along those vectors, generating a posterior probability distribution of the predicted differences of the total effects between the two treatments (see electronic supplementary material) [22]. Here, we indexed parameters according to tail treatments (intact versus tailless), with wing area as a fixed effect and trial day as a random effect, to determine the distribution of mean differences in the probability of being the first moth model attacked. Priors were standardized across mean wing area for all treatments using a normal distribution with an s.d. of 5. We chose 5 as an s.d. based on prior predictive simulation (pps range = 18.7, 46.9). We conducted these analyses in R via the package ‘rstan’ [23]. Statistical and simulation protocols can be found in McElreath [24] and the R code can be found in the electronic supplementary material.

## 3. Results

We found no evidence that tails alter the likelihood of detection and attack by visual predators, compared with no tails. Wrens did not show preferential attack towards one or the other treatment (% first attack on tailed = 0.51 ± 0.08, % first attack on tailless = 0.49 ± 0.08). Side of the platform on which the treatments were presented, treatment of moth first struck the previous day, moth surface area, and whether birds completed this or another behavioural paradigm first, were all uninformative covariates (*p* > 0.05) (see electronic supplementary material for parameter estimates). Our counterfactual simulations support these outcomes across 10 000 iterations: the probability of being attacked first by a foraging bird was equal across tailed and tailless models (mean probability of difference = −0.009, credible interval: −0.41–0.41), while accounting for moth model size and trial day (see electronic supplementary material for full model outputs). Moth surface area was not significantly different between the two treatments (surface area tailed = 35.17 ± 6.1 cm^2^, surface area tailless = 30.95 ± 3.9 cm^2^).

Nearly all birds did not consume the entire dough body, but rather pecked at it or dug out the mealworms encased within the dough. We do not consider this lack of enthusiasm for the dough to be of major concern, however, given that birds were only tested with moth models twice and thus did not have time to develop a specific affinity or aversion to the dough body (as evidenced by no significant shift in attack behaviour between day 1 and day 2 of the assay).

## 4. Discussion

Foraging birds did not demonstrate any difference in initial strikes against moths with or without tails. We therefore do not find evidence that elaborate hindwing tails make roosting moths more conspicuous. It is possible that the foraging task was too simple for the birds, obscuring any subtle differences in diurnal predator detection. While this may be the case, birds did not always seem to notice moth models right away, with multiple individuals (*n* = 7) taking over half an hour to peck at either moth model. For birds that did recognize and attack moth models quickly, it could not be determined from videos whether they had visually detected and made a foraging decision before landing on the platform, as the camera was focused on this interaction space, but in nearly half of all trials (19/43) the bird did not attack the moth model closest to its initial landing spot on the platform. It is also possible that we did not find a difference in detection or strike times between the two treatments because the birds were not under time pressure. During both acclimation and trial days, birds had multiple hours to consume their food allotment. Moreover, while the flight cages were outside in a natural area and therefore were likely exposed to wild predator cues, wrens may have gotten a sense that the cage afforded them safety from predation, further limiting their drive to make quick foraging decisions. It may be that without a sense of constrained foraging time, we could not measure the speed–accuracy trade-off costs that would be apparent in a wild setting [25].

In a natural context, when an animal's attention is divided among multiple tasks, it may rely on search image formation to find prey items more efficiently [26]. A study into the perceptual mechanism underlying search images in birds revealed that this phenomenon is created by a focus on certain features that can be used to separate the prey object from the background, rather than an internal template image of the prey, as had previously been suggested [27]. While tails are obvious to human observers, they may not be as salient to birds and thus may not be one of the traits birds use to distinguish the prey from the leaf background. A similar result was found in a landscape-scale study with fairy wrens, testing what is commonly assumed to be a trade-off—bright plumage in males. These authors found that models with more conspicuous plumage were not attacked at a greater frequency than duller-coloured models [28], highlighting the importance of studying ecological costs of apparently conspicuous traits.

Our results suggest that nocturnal, rather than diurnal, predation is a major driver of hindwing tail evolution in saturniid moths, without clear countervailing costs from birds during the day. Tails are effective against bats, with increasingly long tails leading to increasingly successful deflection, drawing the attack away from the moth's body [10]. A recent study of *Iphiclides podalirius* swallowtail butterflies indicates that their tails deflect bird attack in flight, mirroring the function of moth tails at night against echolocating predators [29]. This deflection phenomenon is also reminiscent of lizard and lycaenid butterfly tails that seem to draw bird strike once the attack is underway [30,31]. It may be that trailing appendages are effective at re-directing predators once the prey animal has been detected, and especially once it is fleeing, but that they do not serve the same purpose in a static position. Alternatively, tails may play a different role against other visual predators (lizards, wasps, etc. [32]), although this will require future behavioural testing.

Without diurnal trade-offs, the evolutionary pressure on hindwing tails by nocturnal predators could lead to nearly Fisherian-esque elaboration of the trait [33]. While tails have originated multiple independent times across the Saturniidae family, they are not the norm and aside from *Actias,* tailed genera are not as speciose as many other non-tailed lineages [10,11]. The origin and proliferation of tails could be limited by other pressures, including flight mechanics [34,35], thermoregulation [36] or pupal metabolic costs [37]. More work parameterizing the flight kinematics of naturally tailed versus non-tailed moths in flight, as well as pre-flight warm-up time or pupal development energetics, etc., will help elucidate the physical limits of tail elongation. Without clear constraint, we hypothesize that lineages in which tails originate might experience a consistent and swift evolutionary trend toward the exaggeration of this trait. This study demonstrates that elaborated traits shaped by one predatory force do not necessarily come at the cost of another and highlights the importance of empirically examining the suite of evolutionary forces constraining and maintaining a trait.

## Data Availability

The data and code are provided as electronic supplementary material [38].
